# Reevaluation of Acromegalic Patients in Long-Term Remission according to Newly Proposed Consensus Criteria for Control of Disease

**DOI:** 10.1155/2014/581594

**Published:** 2014-12-21

**Authors:** Elisa Verrua, Emanuele Ferrante, Marcello Filopanti, Elena Malchiodi, Elisa Sala, Claudia Giavoli, Maura Arosio, Andrea Gerardo Lania, Cristina Lucia Ronchi, Giovanna Mantovani, Paolo Beck-Peccoz, Anna Spada

**Affiliations:** ^1^Endocrinology and Diabetology Unit, Fondazione IRCCS Ca' Granda Ospedale Maggiore Policlinico, Padiglione Granelli, Via F. Sforza 35, 20122 Milan, Italy; ^2^Department of Clinical Sciences and Community Health, University of Milan, 20122 Milan, Italy; ^3^Unit of Endocrine Diseases and Diabetology, Ospedale San Giuseppe Multimedica, 20123 Milan, Italy; ^4^Endocrine Unit, IRCCS Humanitas Clinical Institute, 20089 Rozzano, Italy; ^5^Department of Medicine Biotechnology and Translational Medicine, University of Milan, 20122 Milan, Italy; ^6^Endocrine and Diabetes Unit, Department of Internal Medicine I, University Hospital of Würzburg, 97070 Würzburg, Germany

## Abstract

Acromegaly guidelines updated in 2010 revisited criteria of disease control: if applied, it is likely that a percentage of patients previously considered as cured might present postglucose GH nadir levels not adequately suppressed, with potential implications on management. This study explored GH secretion, as well as hormonal, clinical, neuroradiological, metabolic, and comorbid profile in a cohort of 40 acromegalic patients considered cured on the basis of the previous guidelines after a mean follow-up period of 17.2 years from remission, in order to assess the impact of the current criteria. At the last follow-up visit, in the presence of normal IGF-I concentrations, postglucose GH nadir was over 0.4 *μ*g/L in 11 patients (Group A) and below 0.4 *μ*g/L in 29 patients (Group B); moreover, Group A showed higher basal GH levels than Group B, whereas a significant decline of both GH and postglucose GH nadir levels during the follow-up was observed in Group B only. No differences in other evaluated parameters were found. These results seem to suggest that acromegalic patients considered cured on the basis of previous guidelines do not need a more intensive monitoring than patients who met the current criteria of disease control, supporting instead that the cut-off of 0.4 mcg/L might be too low for the currently used GH assay.

## 1. Introduction

The normalization of growth hormone (GH) and insulin-like growth factor I (IGF-I) secretion is the goal treatment of acromegaly, since it correlates with reduced mortality and morbidity risk, as indicated by a large body of epidemiological data [1–4]. However, different insidious issues concerning broad normal ranges, assay performance, and individual factors, such as age, gender, or BMI, have challenged the identification of definitive guidelines criteria [5, 6]. In effect, the definition of normative reference ranges as well as the criteria for disease remission that best correlate with morbidity and mortality in acromegaly has been an area of debate [2, 4, 7].

Indeed, the availability of high purity of the most recent GH International Standard preparations used for assay calibration, in conjunction with improved IGF-I International Standard [8], together with the assessment of the postglucose GH nadir levels obtained during oral glucose tolerance test (OGTT) in healthy subjects, has led us to define progressively more strict cut-off. Accordingly, the Acromegaly Consensus Group recently revisited the guidelines on criteria for disease control as defined in Cortina in 2000 and recommended a regular retesting in patients with normal age-adjusted IGF-I levels and postglucose GH nadir levels after OGTT ≥ 0.4 mcg/L, obtained using assays that contain standards calibrated against the WHO 2nd International Standard (IS) 98/574, with antibodies which essentially recognize the 22 kDa isoform and with results expressed as mass unit (*μ*g/L) [8–10].

In a previous study carried out on patients defined in remission based on postglucose GH nadir levels < 1 *μ*g/L (obtained with GH assays standardized to the previously adopted IS, i.e., code 80/505) together with normal IGF-I levels, we did not find any differences in terms of biochemical, clinical, and neuroradiological recurrence between acromegalic patients with postglucose GH nadir levels above and below an arbitrarily defined tight cut-off of 0.26 *μ*g/L, that is, mean + 2SD of healthy controls, suggesting that lowering of cut-off postglucose GH nadir values did not improve the discrimination of long-lasting disease remission [11].

Aim of the study was to reevaluate, after a long-term follow-up period (mean: 17.2 ± 8.4 years, range: 3–35 yrs.) and using the latest recommended International Standard for GH and IGF-I assays, a cohort of acromegalic patients that we considered cured on the basis of the previous guidelines to assess disease status according to the new consensus and, therefore, to compare the clinical characteristics and comorbidities of the two groups of patients (e.g., patients who fulfill the current criteria and patients who do not), in order to define long-term remission.

## 2. Subjects and Methods

### 2.1. Patients

The patients recruited for the study were part of a larger cohort of acromegalic patients who, at the time of the last assessment available with GH assay standardized to IS 80/505, were considered cured according to Cortina Consensus (i.e., normal age-adjusted IGF-I levels and postglucose GH nadir < 1 *μ*g/L) [8], after therapy surgery and/or radiation. Further inclusion criteria used for the selection of patients were as follows: minimum follow-up of 3 years after disease remission, complete pre- and postoperative follow-up status (i.e., clinical, hormonal, and neuroimaging data), and availability of long-term assessment with GH and IGF-I assays standardized to WHO IS 98/574 and IS 02/254, respectively.

Forty acromegalic patients (25 females and 15 males, at diagnosis mean age ± SD 40.4 ± 12.3 yrs., BMI ± SD 27.0 ± 4.3 Kg/m^2^, serum GH ± SD mean 19.9 ± 20.4 ng/mL, serum postglucose GH nadir ± SD 16.0 ± 21.9 ng/mL, and serum IGF-I ± SD 101.0 ± 39.5 nmol/L, 9.6 ± 5.2 SDS), judged cured with the previous criteria, were hence reevaluated after a mean follow-up period of 17.2 ± 8.4 years (range: 3–35 yrs.) using the recommended standardization for GH and IGF-I assays.

Hormonal and clinical characteristics of 16 out of 40 patients were previously described [11]. At diagnosis, a pituitary microadenoma was documented in 15 patients and 25 patients had a macroadenoma, with an extrasellar extension in 20. Patients treated with neurosurgery alone represented the majority of our series (75%) at long-term follow-up, whereas in 10 cases surgery was followed by radiotherapy when appropriate (6 by conventional fractionated radiotherapy and 4 by Gamma Knife). Sixteen of 40 patients showed isolated or multiple pituitary failures that were adequately substituted (10: hypoadrenalism, 2: hypothyroidism, and 8: hypogonadism). Pregnant women were excluded and women of fertile age were tested during the early follicular phase of the menstrual cycle (days 1–5); no woman received oral estrogens.

Clinical and hormonal data of patients are summarized in Table 1.

### 2.2. Study Protocol

We used medical records to retrospectively investigate posttreatment outcomes and hormonal, clinical, neuroradiological, and metabolic data for each patient. Acromegalic patients were evaluated in three different time points: at the time of remission (phase 1), at the time of the last available assessment with the GH assay standardized to IS 80/505 (mean 6.9 ± 5.4 years, phase 2), and at the last follow-up visit assessed with the GH assay standardized to IS 98/574 and IGF-I assay standardized to IS 02/254 (mean 17.2 ± 8.4 years after disease remission, phase 3). All patients were evaluated after an overnight fast; blood samples were collected after 1 hour of bed rest with a slow saline infusion for serum GH (mean of at least 3 samples during saline infusion) and IGF-I levels determination. After 75 g oral glucose load, additional blood samples for GH measurements were collected at 30, 60, 90, and 120 minutes. Postglucose GH nadir of individual patient was defined as the lowest GH value at any time during two-hour OGTT.

All patients were studied for metabolic and cardiovascular comorbidities; in particular, glucose and insulin levels at baseline and after 120 minutes during 2 h OGTT, insulin resistance by the homeostatic model assessment [HOMA-IR = fasting glucose (mmol/liter) × fasting insulin/22.5] and the quantitative insulin check index QUICKI = 1/[log FG (mg/dL) + log FI (mU/liter)], glycosylated haemoglobin (HbA1c), systolic and diastolic blood pressure (SBP and DBP) measured according to the World Health Organization (WHO) International Society of Hypertension Guidelines, lipid profile encompassing total cholesterol (TC), high density lipoprotein cholesterol (HDL), and triglycerides (TG) were evaluated. Low density lipoprotein cholesterol levels (LDL) were evaluated by the formula LDLc = TC-HDLc-TG/5. Patients were considered affected with hypertension by the presence of systemic blood pressure ≥140 mmHg or diastolic blood pressure ≥90 mmHg or use of antihypertensive therapy, while glucose tolerance was defined according to accepted international criteria. In addition, body mass index (BMI) and waist circumference (WC) were evaluated.

They were also investigated for clinical symptoms and signs of disease (e.g., arthralgia, hyperhidrosis, soft tissues swelling, paraesthesias, headache and asthenia, and the fourth finger size of left hand by jewellery rings), for pituitary function and adequacy of replacement therapy. In a day different from that of OGTT, a GHRH plus arginine test for the evaluation of GH peak was performed in order to assess the residual GH secretion (GHRH 1–29; GEREF, Serono: 1 *μ*g/Kg i.v. at 0 min.; arginine hydrochloride, 0.5 g/Kg i.v. over 30 min. from 0 to 30 min. up to a maximum of 30 g); however, since the assessment of residual GH secretion was not a primary objective of the study, the stimulation test was performed in 22 out of 40 patients (55%), randomly selected in both groups. Blood samples for GH evaluation were taken at 0, 15, 30, 45, 60, 90, and 120 min. According to the more recent criteria, GH deficiency (GHD) has been defined by GH peak < 11.5 *μ*g/L if BMI was below 25 Kg/m^2^, <8.0 *μ*g/L if BMI was between 25 and 30 Kg/m^2^, and <4.2 *μ*g/L if BMI was over 30 Kg/m^2^ [12].

The occurrence of cardio- and cerebrovascular disease (e.g., cardiomyopathy, valvular disease, angina, acute myocardial infarction, and stroke), malignancies, and colorectal disease (e.g., colonic diverticula, dolichocolon, and colonic polyps) was investigated as well; smoking habits were also assessed during the follow-up, as a possible confounding factor.

Serum samples were kept frozen at −20°C until assayed. All hormone determinations were performed in the same local laboratory using the same assays.

In addition, a magnetic resonance imaging (MRI) of the sellar region before and after gadolinium contrast was performed in all patients at last follow-up visit and it was evaluated by the same neuroradiologist, with the aim of assessing the presence of potential recurrence or regrowth with respect to the early postoperative imaging (CT or MRI, where available).

The Local Ethical Committee (Fondazione IRCCS Ca' Granda, Milan) approved the protocol study and patients gave their informed written consent to participate in the study.

### 2.3. Hormonal Assays

Until 1992, serum GH levels were measured with radioimmunoassay (RIA) with polyclonal Lisophase HGH kit (Sclavo, Milan, Italy). The sensitivity of the method was 0.3 mg/L and intra- and interassay coefficients of variation were both below 10%. The standards were calibrated to the first World Health Organization International Reference Preparation HGH-MCR (code 66/217). Subsequently and prior to October 2007, a two-site monoclonal immunofluorometric assay method (AutoDelfia kit, Wallac, Inc. OY, Turku, Finland) was used. The sensitivity of this method was 0.01 mg/L and intra- and interassay coefficients of variation were 2% and 1.7%, respectively. After October 2007, GH was assayed with a chemiluminescence method (Immulite 2000, Siemens Medical Solutions Diagnostics, Los Angeles, CA) with a detection limit of 0.01 mg/L. In both cases, the standards were calibrated to the first World Health Organization International Reference Preparation (code 80/505). After the second semester of 2010, the standards were calibrated to the WHO International Standard IS 98/574.

Serum IGF-I concentrations were measured by commercial RIA kits starting from 1985. According to the RIA assay used before 1996 (Incstar, Stillwater, MN), the removal of binding proteins was obtained by acidification and subsequent filtration on ODS C18 cartridges. The intra- and interassay coefficients of variation were 15 and 16%, respectively. Afterwards and until 2008, IGF-I levels were assessed by the commercial radioimmunometric assay kit of Mediagnost (Tübingen, Germany). The separation of IGF-I from binding proteins was obtained by acidification in IGF-II excess, and IGF-II cross-reactivity was less than 0.05%. The intra- and interassay coefficients of variation were 3.2 and 8.9%, respectively. After 2008, IGF-I levels were measured by a chemiluminescent immunometric assay (Immulite 2000 IGF-I; Siemens Medical Solutions Diagnostics, Los Angeles, CA), with an intra- and interassay coefficient of variation of 2.9 and 7.4%, respectively. The values were compared with those from an appropriate age- and sex-adjusted range and expressed in standard deviation scores (SDS), as recently suggested.

All other biochemical and hormonal parameters (e.g., glucose, insulin, and prolactin) were measured by standard procedures.

### 2.4. Statistical Analysis

Results were expressed as mean ± SD, unless otherwise stated. A paired Student's* t*-test was performed to compare different variables in case of normally distributed data; non-Gaussian variables were compared using Wilcoxon-Mann-Whitney test.

Three groups comparisons were carried out by ANOVA with Bonferroni post hoc test. In case of nonnormal variables, a nonparametric Kruskal-Wallis test was used. Fisher's exact test was used to compare number of patients in 2 × 2 contingency tables. A backward stepwise logistic regression model was used to analyse the possible role of clinical and hormonal variables in determining postglucose GH nadir classification (<0.4 *μ*g/L or ≥0.4 *μ*g/L). *P* > 0.10 was used as removal criterion.

Values of *P* < 0.05 were considered statistically significant.

Analysis was performed by IBM SPSS statistic software, version 20.0.

## 3. Results

### 3.1. Hormonal and Metabolic Data of the Cohort of Acromegalic Patients during Long-Term Follow-Up

Mean serum GH levels in basal condition were 0.9 ± 1.2 *μ*g/L at long-term follow-up from disease remission (mean 17.2 ± 8.4 yrs., phase 3), without significant reduction compared to phase 1 and phase 2 (Table 1). At last follow-up visit, IGF-I levels were below the upper limit of normal range in all patients, with a significant decrease if compared to early postoperative phase (−0.28 ± 1.1 versus 0.6 ± 1.9 SDS, *P* = 0.02, Table 1).

At the long-term follow-up visit, mean postglucose GH nadir was 0.22 ± 0.20 *μ*g/L (range: 0.01–0.99), with a significant reduction with respect to phase 1 (0.22 ± 0.20 versus 0.39 ± 0.38 *μ*g/L, *P* < 0.05, Table 1). In particular, female patients and those with macroadenomas demonstrated a trend towards lower postglucose GH nadir during long-term follow-up (Table 1). No significant correlations between mean postglucose GH nadir and IGF-I SDS, sex, age, BMI, radiotherapy, and tumor dimension were found (data not shown). Finally, no correlation between the postglucose GH nadir values and the presence of GH deficiency or other pituitary failure was detected.

Overall, no significant differences in any of the evaluated metabolic parameters (i.e., fasting and 120 min glycemia, fasting and 120′ min insulinemia, total cholesterol, triglycerides, HDL-cholesterol, HOMA-IR index, QUICKI, BMI, waist circumference, and systolic and diastolic blood pressure) were observed at long-term follow-up compared to early postoperative period (data not shown).

### 3.2. Comparison of Hormonal, Metabolic, and Comorbidity Data between Patients with Postglucose GH Nadir over (Group A) and below 0.4 *μ*g/L (Group B) during Long-Term Follow-Up

**Figure 1:**
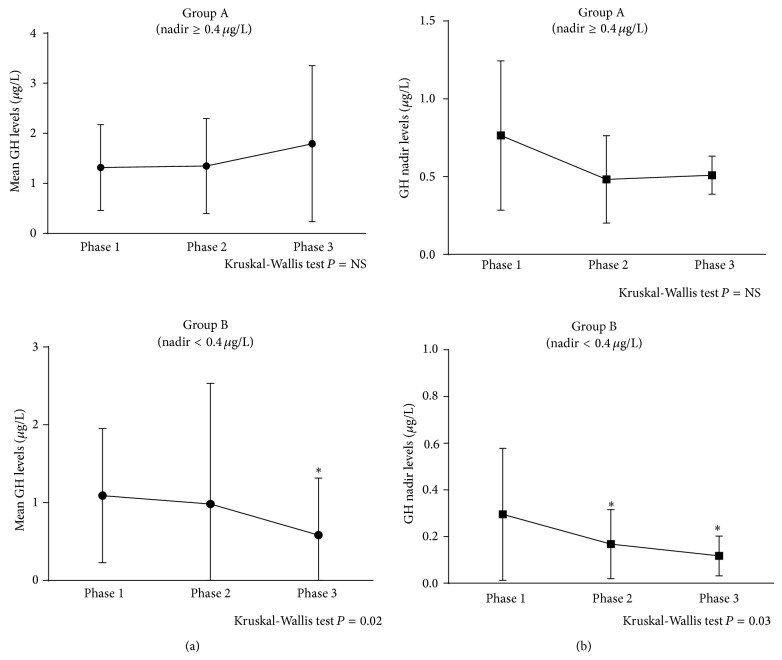
(a) Mean GH levels in basal condition, evaluated as the mean of at least three consecutive samples, in patients of group A (i.e., GH nadir ≥ 0.4 mcg/L, upper) and group B (i.e., GH nadir < 0.4 mcg/L, lower). Patients of group B showed mean GH levels at long-term follow-up (phase 3) significantly lower than at the time of remission (phase 1), whereas no difference was observed in group A. ^*^ *P* < 0.05 versus phase 1. Values are expressed as mean ± SEM. (b) Mean GH nadir, defined as the lowest GH value at any time after glucose administration (2 h-OGTT), in patients of group A (i.e., GH nadir ≥ 0.4 mcg/L, upper) and group B (i.e., GH nadir < 0.4 mcg/L, lower). Patients of group B showed mean GH nadir levels atlong-term follow-up (phase 3) and at the time of the last assessment available with the GH assay standardized to IS 80/505 (phase 2) significantly lower than at the time of remission (phase 1), whereas the decrease did not reach statistical significance in patients of group A. ^*^ *P* < 0.05 versus phase 1. Values are expressed as mean ± SEM.

Considering the current criteria for curing acromegaly, postglucose GH nadir levels over the cut-off limit of 0.4 *μ*g/L were observed in 11 out of the whole cohort of 40 patients (28%) in the presence of normal IGF-I concentrations (group A, 8F&3M, mean age 58.0 ± 7.9 yrs., 3 pts. previously irradiated), whereas the remaining 29 patients had postglucose GH nadir levels below 0.4 *μ*g/L (group B, 17F&12M, mean age 54.3 ± 12.4 yrs., 7 pts. previously irradiated). No statistical difference in IGF-I SDS levels at diagnosis, phases 1 and 2, was found between the two groups (Table 2). Conversely, mean basal GH levels that were evaluated by multiple sampling as suggested by the recent consensus criteria in case of discrepant biochemical results showed a tendency to be higher in group A than in group B, a tendency that reached the statistical significance at the last follow-up (Table 2). During the whole follow-up period, the number of patients of the two groups who showed GH levels greater than safe value of 2.5 *μ*g/L was comparable (Table 2). However, mean GH and postglucose GH nadir levels at last follow-up (phase 3) were significantly lower than phase 1 only in patients of group B (i.e., postglucose GH nadir < 0.4 *μ*g/L, resp., *P* = 0.03 and *P* = 0.002), whereas the decrease did not reach statistical significance in patients of group A (Figure 1). The dynamic evaluation of residual GH secretion performed in 22 out of 40 patients (55%), randomly selected in both groups, showed that the number of patients with GH deficiency was not significantly different in group A and group B (data not shown).

No significant differences in the other evaluated parameter (sex, age, timing of follow-up, time of postglucose GH nadir, tumor size, and radiotherapy) were found between the two groups (Table 2). In addition, no potential recurrences or regrowth was detected at last follow-up MRI evaluation in any patients. Finally, both logistic and linear backward stepwise regression analyses were carried out to evaluate the impact of the following predictors: gender, age, adenoma size, BMI, radiotherapy, preoperative IGF-I SDS, preoperative mean GH, and postglucose GH nadir levels, on long-term follow-up postglucose GH nadir levels. None of these factors resulted to be a significant independent predictor of higher postglucose GH nadir.

Interestingly, as far as metabolic data and comorbidities of acromegaly were concerned, no differences were evidenced between group A and group B (Table 3). Finally, the number of patients who smoke-assessed during the follow-up as a possible confounding factor, symptom score, ring size, and pituitary function were not significantly different between group A and group B (data not shown).

## 4. Discussion

The consensus guidelines updated in 2010 revisited the remission criteria in acromegalic patients treated with neurosurgery, defining “disease control” as postglucose GH nadir levels below 0.4 *μ*g/L and IGF-I levels in age-adjusted normal range, both dosed with ultrasensitive methods [9]. If these criteria are applied, it is likely that a percentage of patients who were previously classified as cured might present postglucose GH nadir levels not adequately suppressed, with potential implications for the management of the follow-up of these patients.

The present study explored GH secretion, using the latest International Standards recently recommended (i.e., code 98/574 for GH assay and code 02/254 for IGF-I assay), as well as hormonal, clinical, neuroradiological, metabolic, and comorbidities profile in a cohort of 40 acromegalic patients considered cured on the basis of the previous guidelines, after a very long-term follow-up period, in order to assess the impact of the recently proposed criteria for postglucose GH nadir values to define the postoperative long-term remission and a treatment as much as possible personalized.

The main result was that, after a median long-term follow-up of 17.2 years from disease remission, no difference was found in any of the evaluated parameters between patients with postglucose GH nadir levels over or below 0.4 *μ*g/L, using ultrasensitive assays.

In particular, in all patients with postglucose GH nadir over 0.4 *μ*g/L but below 1 *μ*g/L (group A), who were considered as cured so far on the basis of the previous guidelines, IGF-I levels persisted in the normal age-adjusted range throughout the follow-up evaluation.

In addition, patients of group A, representing 28% of patients of the present series, did not have a higher incidence of endocrine and metabolic complications or other comorbidities in the long-term follow-up. Finally, none of the factors considered (i.e., gender, age, adenoma size, preoperative IGF-I SDS, preoperative mean GH levels, and GH nadir after glucose load and radiotherapy) resulted to be a significant independent predictor of higher postglucose GH nadir levels by logistic regression analysis.

Despite the lack of clinical differences between the two groups, we observed that the profile of the mean GH levels and postglucose GH nadir was slightly different in the two groups at last follow-up. In fact, mean GH levels resulted to be significantly lower in group B than in group A and, in parallel, there was a progressive reduction of both mean GH levels and postglucose GH nadir values only in group B, while the IGF-I levels have remained broadly stable and overlapping postoperatively in both groups. In addition, we found that the incidence of mean GH levels above the safe cut-off of 2.5 *μ*g/L was, however, comparable. In this respect, it is tempting to speculate that, in analogy with the reduction of the GH nadir values, the cut-off of 2.5 *μ*g/L for mean safe GH should be revised in the light of the ultrasensitive assays.

It is at present difficult to ascertain whether this decline in GH levels might represent a sign of normalization of GH secretion pattern or, instead, of GH deficiency that frequently occurs in cured acromegalic patients [13, 14]. A possible bias in the lack of correlation between GHD and postglucose GH nadir levels might be related to the fact that only one-half of the patients were tested for GHD with dynamic stimulation in the present study.

In the attempt to correlate IGF-I concentrations with GH secretion and to explore the significance of divergent GH/IGF-I profiles, the role of adjuvant radiotherapy has been investigated as well. It is known that irradiation seemed to imply a flat GH secretory pattern, with a less pronounced effect on the normalization of IGF-I; in addition, identical degrees and rates of GH decline have been observed in relationship with different doses administered and in the techniques employed [15, 16]. In our study, radiotherapy (conventional fractionated radiotherapy and Gamma Knife) was equally distributed between group A and group B at the last follow-up visit and we did not observe significant differences in postglucose GH nadir levels between irradiated patients and those not treated with radiotherapy. Otherwise, we found that mean basal GH levels were significantly decreased at last follow-up versus phase 1 and phase 2 only in irradiated patients, when the acromegalic patients were considered all together.

A clinical interpretation of our results could arise from the awareness of the problem of GH assay variability: as Bidlingmaier suggested, several problems with GH assays cannot be solved solely by using a single calibrator for all assays and probably the need for strictly “method specific cut-off values,” advocated from most guidelines to improve reliability, should be object of collaborative efforts [17]. In this regard, Arafat and his colleagues have shown that using Immulite 2000 results are about 2.3 times higher than the dose Nichols and 6 times higher than the Diagnostic System Laboratories, even when the recalibration has been taken into account, and have identified a cut-off assay-specific GH nadir equal to 1 mcg/L, able to identify the 78–80% of patients in remission and 95% of patients with active disease [6]. Furthermore, a recent publication of Müller and colleagues has confirmed that the Immulite GH assay, even after Siemens claimed to have recalibrated the assay against the new recombinant standard (i.e., 98/574), still gives GH concentrations considerably higher than those obtained by all other assays [18].

Secondly, the present findings seem to corroborate some previous studies showing that, in presence of discrepant results, IGF-I may have greater prediction power over postglucose GH nadir values [2, 11, 19], even though some limitations of IGF-I parameter cannot be neglected, such as binding proteins interference and inadequate international reference standards and reference values [20]. In the past, subtle abnormalities of GH suppression in patients with normal IGF-I values have been extensively investigated. In particular, it has been suggested that incomplete GH suppression during OGTT might indicate persistence of postoperative central deregulation and not necessarily GH hypersecretion, while IGF-I levels might remain normal even in the presence of mild GH excess [19, 21]. In a previous work, we demonstrated that defective GH suppression to glucose is unlikely to be due to functional and/or anatomical hypothalamic-pituitary disconnection, since a normal postglucose GH nadir was recorded in patients with various hypothalamic-pituitary disorders other than acromegaly [22].

The relatively low number of subjects evaluated in our study and the variability of factors that may influence GH levels require a degree of caution in reaching final conclusion, even though the use of the same central well-validated GH and IGF-I assays in all patients represents a strength that enables a confident reading of our main findings. Nevertheless, the very long-term follow-up of these acromegalic patients, considered cured on the basis of the previous guidelines, seems to demonstrate that they do not need a closer or more intensive monitoring than patients that met the current criteria of disease control (e.g., postglucose GH nadir less than 0.4 mcg/L and normal age-adjusted IGF-levels), suggesting instead that the cut-off of 0.4 mcg/L might be too low for the currently used GH assay. Indeed, according to the clinical context, a periodical long-term monitoring of both GH and IGF-I levels is required in acromegalic patients, in reason of either GH deficiency occurrence or possibly disease recurrence.

## Figures and Tables

**Table 1:** Pre- and postoperative hormonal data of overall acromegalic patients (*n* = 40) during longitudinal follow-up; postglucose GH nadir levels are additionally categorized by sex and tumor size. Mean ± SD values are shown.

Parameter	Preoperative	Phase 1	Phase 2	Phase 3
Mean (years)	—	0.7 ± 0.4	6.9 ± 5.4	17.2 ± 8.4
Age (years)	40.4 ± 12.3	45.1 ± 11.3	50.7 ± 11.0	55.4 ± 11.3
IGF-I (SDS)	9.6 ± 5.2	0.6 ± 1.9	−0.32 ± 0.9	−0.28 ± 1.1^■^
Basal GH (*μ*g/L)	19.9 ± 20.4	1.1 ± 0.9	1.0 ± 1.4	0.9 ± 1.2
Range	(1.0–73.0)	(0.0–3.0)	(0.0–7.0)	(0.0–4.4)
Nadir GH (*μ*g/L)	16.0 ± 21.9	0.39 ± 0.38	0.27 ± 0.29	0.22 ± 0.20^■^
Female (*n* = 25)	19.6 ± 27.7	0.45 ± 0.42	0.27 ± 0.25	0.24 ± 0.21^■^
Male (*n* = 15)	11.2 ± 9.5	0.30 ± 0.30	0.29 ± 0.37	0.21 ± 0.19
Micro (*n* = 15)	7.2 ± 6.0	0.27 ± 0.29	0.22 ± 0.27	0.18 ± 0.18
Macro (*n* = 25)	27.0 ± 28.5^a^	0.51 ± 0.42	0.31 ± 0.31	0.25 ± 0.21^■^

Phase 1: at the time of remission.

Phase 2: at the time of the last available assessment with the GH assay standardized to IS 80/505.

Phase 3: after a long-term follow-up with ultrasensitive GH assay standardized to IS 98/574.

Micro: microadenoma; macro: macroadenoma.

^■^ *P* < 0.05 versus phase 1.

^a^ *P* < 0.05 versus microadenoma.

**(a):** 

Parameters	Group A	Group B	
	(GH-n ≥ 0.4)	(GH-n < 0.4)	
*n*	11	29	—
Sex (F/M)	8/3	17/12	ns
RT (y/n)	3/8	7/22	ns
Yrs. since disease remission	19.8 ± 9.7	15.1 ± 7.5	ns

**(b):** 

	Preoperative	Phase 1	Phase 2	Phase 3	Preoperative	Phase 1	Phase 2	Phase 3
Basal GH (*μ*g/L)	25.9 ± 23.3	1.3 ± 0.9	1.3 ± 1.0	1.8 ± 1.6^*^	18.2 ± 19.6	1.0 ± 0.9	0.9 ± 1.5	0.6 ± 0.7^■^

Mean basal GH >2.5 *μ*g/L (y/n)	—	2/9	1/10	3/8	—	3/26	2/27	1/28

Postglucose nadir GH (*μ*g/L)	9.5 ± 6.5	0.76 ± 0.48^*^	0.48 ± 0.28^*^	0.51 ± 0.11^*^	13.7 ± 13.5	0.30 ± 0.28	0.17 ± 0.15^■^	0.11 ± 0.08^■^

Time of postglucose GH nadir (min)	—	—	—	62.7 ± 36.6	—	—	—	63.8 ± 31.0

IGF-I (SDS)	12.0 ± 6.8	0.4 ± 1.7	−0.7 ± 0.9	−0.4 ± 1.0	9.1 ± 4.7	0.6 ± 2.0	−2.8 ± 14.8	0.2 ± 1.7

F/M: female/male; RT: radiotherapy; y/n: yes/no; yrs.: years; GHD: GH deficiency.

Phase 1: at the time of remission.

Phase 2: at the time of the last available assessment with the GH assay standardized to IS 80/505.

Phase 3: after a long-term follow-up with ultrasensitive GH assay standardized to IS 98/574.

^*^ *P* < 0.05 versus corresponding phase group B.

^■^ *P* < 0.05 versus phase 1 same group.

**Table 3:** Comparison of metabolic and comorbid data between patients with postglucose GH nadir over (group A) and below 0.4 *μ*g/L (group B) at last follow-up.

Parameter	Group A	Group B	*P*
	(GH-n ≥ 0.4 mcg/L)	(GH-n < 0.4 mcg/L)	
*n*	11	29	—
BMI (Kg/m^2^)	24.4 ± 4.6	28.6 ± 4.4	ns
Impaired glucose metabolism (y/n)	4/7	13/16	ns
IFG, IGT, DM (n)	1/2/1	0/9/4	
Fasting glucose (mg/dL)	85 ± 14	97 ± 20	ns
Glucose at 120′ (mg/dL)	138 ± 57	128 ± 36	ns
HOMA-IR	2.0 ± 1.7	2.1 ± 1.8	ns
Blood hypertension (y/n)	3/8	12/17	ns
SBP (mmHg)	115 ± 15	122 ± 12	ns
DBP (mmHg)	74 ± 11	79 ± 7	ns
Cardio- and/or cerebrovascular disease (y/n)	6/5	10/19	ns
Valvular heart disease	4	4	
Acromegalic cardiomyopathy	1	1	
Arrhythmias and cardiomyopathy	0	5	
Acute myocardial infarction	1	0	
Malignancies (y/n)	3/8	2/27	ns
Mammary carcinoma	1	0	
Monoclonal gammopathy	2	0	
Olfactory meningioma	0	1	
Skin melanoma	0	1	
Colorectal disease	2/9	7/22	ns
Diverticula	0	3	
Dolichocolon	0	1	
Hyperplastic polyps	0	2	
Adenomatous polyps	2^*^	1°	

IFG: impaired fasting glucose; IGT: impaired glucose tolerance; DM: diabetes mellitus; NS: not significant.

^*^Low grade adenomatous tubular polyps, °serrated adenoma with low grade dysplasia.
